# Paediatric Boerhaave’s syndrome: a case report and review of the literature

**DOI:** 10.4076/1757-1626-2-8302

**Published:** 2009-08-20

**Authors:** Maruthesh Gowda Chikkappa, Charles Morrison, Andrew Lowe, Shaun Gorman, Ralph Antrum, Jay Gokhale

**Affiliations:** Department of General Surgery, Bradford Royal InfirmaryBradford, BD9 6RJUK

## Abstract

We report a case of paediatric Boerhaave’s syndrome in 15-year-old girl associated with massive dilatation of the stomach into the pelvis and transient hepatitis of uncertain aetiology. This cluster of clinical finding has not previously been reported. The young girl initially presented with abdominal pain, vomiting and lower urinary tract symptoms. She was initially treated for urinary tract infection after urine dipstick showed leucocytes and nitrates. Later she was found to have the spectrum of findings as described. Patient was treated by restricting to strict no oral intake and gastric decompression. Enteral nutrition maintained via a feeding jejunostomy.

Boerhaave’s syndrome frequently presents in the context of other emetogenic illnesses which may mimic its features as a result the diagnosis can be difficult. A high index of clinical suspicion is therefore required. We review the literature of paediatric Boerhaave’s syndrome to aid the clinician with this diagnostic conundrum.

## Case presentation

A 15-year-old, Caucasian, British, previously fit and healthy girl presented with a four-day history of abdominal pain and vomiting. The only past medical history was appendicitis, for which she had undergone an uncomplicated laparoscopic appendicectomy at the age of 13 years. In the days preceding admission, she had been suffering from suprapubic pain, dysuria and urinary frequency; associated with nausea and vomiting. On the day prior to hospital admission she had two episodes of vomiting associated with severe epigastric pain for which general practitioner started her on Cefalexin on the basis of urinalysis which was later confirmed on microscopy. She was admitted to the hospital the following day with worsening symptoms.

On arrival she was tachycardic with a pulse 151 beats per minute and had low grade pyrexia of 37.4°C. Her abdomen was mildly distented and exquisitely tender in the epigastrium. Urine analyses confirmed that she was not pregnant. On investigation she was found to be polycythaemic with haemoglobin of 18 mg/dL. Her leucocyte count was 13 × 10^9^/ml with a neutrophilia of 11 × 10^9^/ml. The serum urea and creatinine levels were elevated at 13.5 mmol/L and 110 µmol/L respectively. The serum bilirubin was 51 µmol/L, although she did not clinically appear jaundiced. Serum aspartate transaminase was also elevated at 227 IU/L but serum alkaline phosphatase was within normal limits, 213 iu/L. Chest radiograph initially reported normal. Hepatitis, CMV and EBV serology later proved to be negative.

The patient was fluid resuscitated and the cefalexin, commenced by the GP, changed to intravenous cefuroxime. At this point, sepsis secondary to urinary tract infection was the working diagnosis. Abdominal imaging was requested to exclude the possibility of cholestasis or choledocholithiasis causing the symptoms of upper abdominal pain and the deranged liver function profile.

Initial imaging with ultrasonography of the abdomen showed no abnormality of the biliary tree or gallbladder. It did however show a hugely dilated stomach extending into the pelvis and dilated proximal duodenum suggestive of distal duodenal obstruction. The cause of the obstruction could not be determined. Stomach was decompressed with a nasogastric tube which drained 1000 ml of bilious fluid immediately.

**Figure 1.**
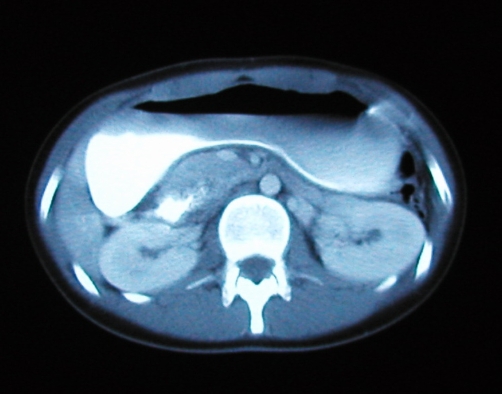
CT showing gastric dilatation.

**Figure 2.**
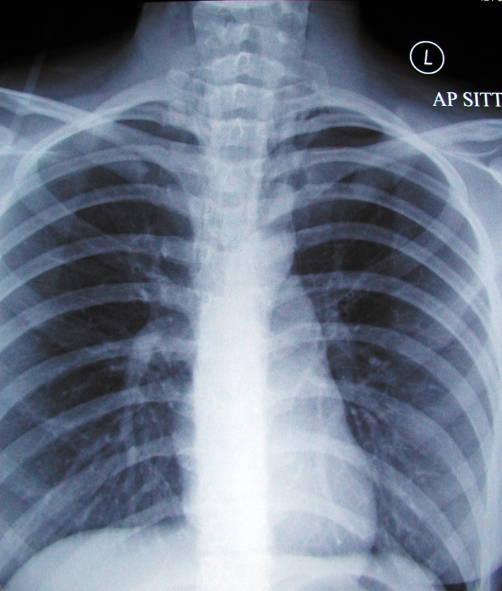
CXR showing surgical emphysema.

**Figure 3.**
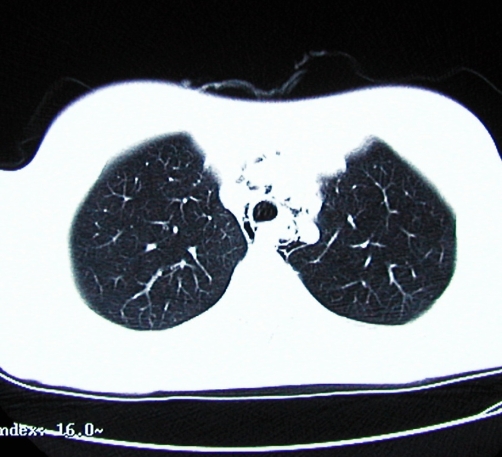
CT showing pneumomediastinum.

**Figure 4.**
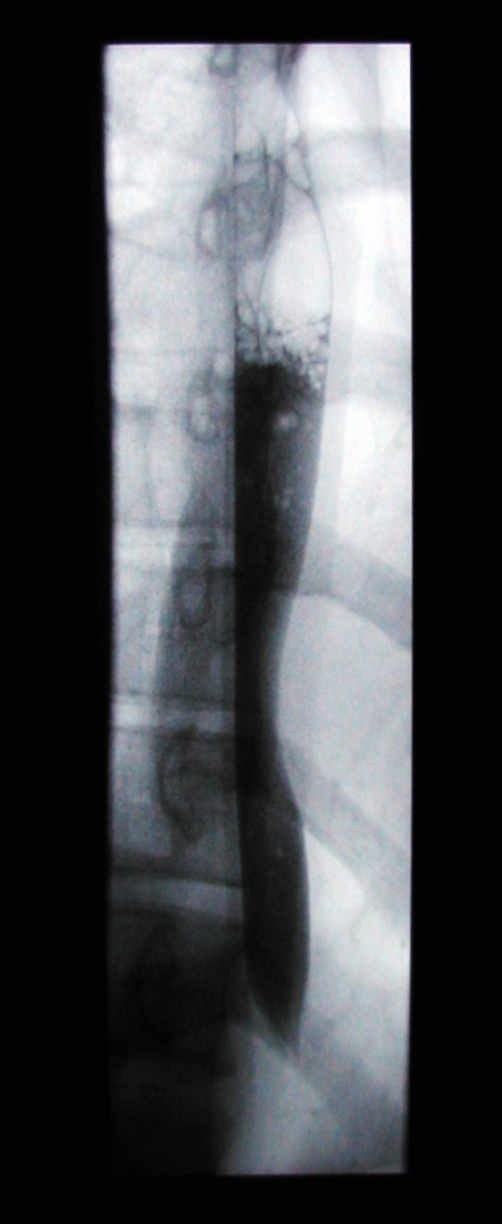
Gastrograffin showing no ongoing leak.

Imaging followed, with computerised tomography (CT) of her abdomen and pelvis, which again demonstrated a hugely dilated stomach (Figure 1) but no mechanical obstruction. It did however show air within the inferior portion of the mediastinum. Retrospective review of her chest radiograph, confirmed the presence pneumomediastinum (Figure 2). This was followed up with CT of the thorax which showed extensive mediastinal gas (Figure 3); suggestive of spontaneous oesophageal rupture; Boerhaave’s syndrome. At this point broad spectrum antibiotic were initiated. Gastrograffin swallow did not demonstrate on going leak (Figure 4) but showed massive gastric dilatation, the cause of which was unclear.

Because of concerns of mechanical obstruction, the patient underwent a laparotomy at which no mechanical obstruction was demonstrated. Stomach was inspected fully including posterior aspect and there was no evidence of perforation. The stomach was moderately distended; but showed no gross pathology and was decompressed by venting gastrostomy. A feeding jejunostomy was placed at the time of operation to allow enteral feeding while the oesophageal perforation was allowed to heal. Retrograde enteroscopy, duodenoscopy and gastroscopy were performed and biopsies taken from small bowel and stomach which were all found to be histologically normal.

Postoperatively the patient made excellent progress and was weaned to a normal diet over a period of a month. Her liver functions parameters also returned to normal over the same period. At 6 months following discharge she had experienced no further symptoms and has gained 5.5 kg in weight.

## Discussion

Spontaneous oesophageal rupture was originally described by Herman Boerhaave in 1724 [1]. It typically occurs in men aged 30-40 years after over indulgence in alcohol or food; but may result from any emetogenic pathology [1]. It must be distinguished from the more common traumatic oesophageal rupture and pathological oesophageal rupture. In Boerhaave’s syndrome, the oesophagus undergoes barotrauma when increased intra-gastric pressure is transmitted to the oesophagus against a closed glottis [2]. The classical presentation is Mackler’s triad, which comprises thoraco-abdominal pain, vomiting and surgical emphysema [3].

Diagnosis of Boerhaave’s syndrome can often be a clinical challenge. The single most important factor is a high index of clinical suspicion. Blood investigations are of limited diagnostic use. Large series in the adult population have shown that the most common finding is leucocytosis. In addition up to 50% of patients are polycythaemic [4]. This is thought to be due to third-space sequestration of fluid which was present in this case. A convincing history, suggestive clinical findings and the demonstration of peri-oesophageal air tracks on radiological imaging (typically computerised tomography) causing pneumomediastinum are sufficient to diagnose Boerhaave’s [5-7].

In our case Boerhaave’s syndrome was only detected on computerised tomography and gastrograffin swallow was normal. Contrast oesophagography has been shown to have a sensitivity consistently reported as 70-75% and hence should not be exclusively used to exclude Boerhaave’s syndrome [5].

Other features of Boerhaave’s syndrome include pneunomthorax, pyothorax and pleural effusions [4]. Massive gastric dilatation or gastroparesis, observed in our case, has not previously been reported in the context of Boerhaave’s syndrome. It may result from trauma to the vagal trunks following oesophageal perforation. It is known that trauma and inflammation causes a vagal neuropraxia impeding gastric emptying and motility [8]. We think this vagal trauma is the cause of gastric dilatation as there was no volvulus of midgut, dudeno-jejunal flexure was normal; there were no bands or adhesions which could have caused volvulus.

**Figure 5.**
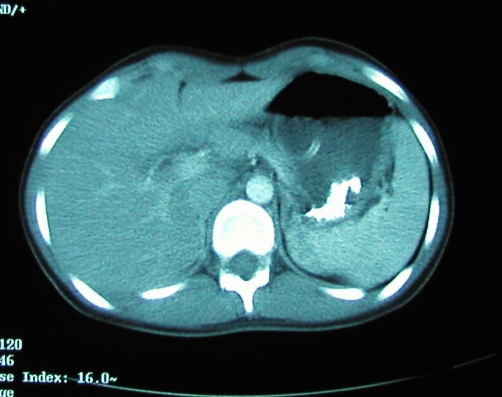
CT confirming hepatitis.

A second feature peculiar to our case was the transient hepatitis (Figure 5) suffered by the patient, underlying cause of which remains unclear. Viral titres were all negative and ultrasonography failed to detect any abnormality in the biliary system or hepatic parenchyma. It may be a feature of the generalised sepsis, related to the cephalosporin administration or simply coincidental.

Boerhaave’s syndrome is very unusual phenomenon in the paediatric population. There are 15 documented paediatric cases [9] out of which 11 are non-neonatal.

Fluid resuscitation and broad spectrum antibiotics are the essential first steps of management. Children tended to do well irrespective of the mode of definitive treatment. Surgical oesophageal repair was performed in five of the eleven non-neonatal paediatric cases. Four were primary procedures and one a delayed procedure [9]. In these five cases there tended to be considerable mediastinal suppuration, hydrothorax and sepsis. The patients were generally clinically very unwell at presentations.

In a large series it was shown that Boerhaave’s is a frequently misdiagnosed condition in the adult population and the correct diagnosis was made within 12 hours in less than 21% of cases [4]. This is somewhat concerning given that its mortality rate makes Boerhaave’s syndrome the most lethal perforation found within the gastrointestinal tract. Although rare, Boerhaave’s syndrome merits consideration in children presenting with thoraco-abdominal pain preceded by vomiting. In younger children presentation is protean; taking a number of forms which may or may not include vomiting as a trigger. A high index of clinical suspicion is required if the diagnosis is not to be missed, particularly as paediatric patients tend to appear much less unwell initially than their adult counterparts.
